# Eraldo Antonini Lectures, 1983–2019

**DOI:** 10.1186/s13062-022-00330-0

**Published:** 2022-07-15

**Authors:** Maurizio Brunori

**Affiliations:** Presidente emerito Classe di Scienze FMN, Accademia Nazionale dei Lincei, e Professore emerito di Chimica e Biochimica, Dipartimento di Scienze Biochimiche, Sapienza Università di Roma, Rome, Italy

**Keywords:** Biochemistry, Molecular biology, Proteins, Protein structure, Allostery

## Abstract

“Can order spring from Chaos?” is the title of an extensive Report on Italian science published by NATURE on 12 May 1983 and written by Robert Walgate, the Chief European Correspondent. It is a twenty pages complete paper touching all aspects of the struggle of Italian scientists to work in the “curious amalgam of ingenuity and muddle, a reflection of the political system”. (Nature, 1983; 303: 109–128). To read it after four decades is interesting but somewhat depressing since the main problems unfolded in the paper have not been solved, starting with the largely insufficient support of fundamental curiosity driven research. At page 114 you could find a item called: ITALY’s TOP SCIENTISTS: Four in the top one thousand. The Author refers to the data reported by the ISI (Institute of Scientific Information) that took two years to scan 3,000 major journals over the period 1965–78 and covered 5 millions articles and 67 millions references. The four top Italian scientists working in Italy were: Eraldo Antonini (3127 citations), Enrico Clementi (4001), Silvio Garattini (2833), and Giorgio Giacomelli (2483); 3 out of four were 52 years old, and one 55. Antonini did not see the Report since he passed away on March 18, 1983. However the information leaked before the publication of Nature because I remember the Messaggero of Rome reporting a whole page with the ranking of the four Italians, and even a picture of Eraldo. The students of the first year Medical course, his Class, welcomed the Professor with a standing ovation. After a short time the Board of the SIB (Società Italiana di Biochimica) casted a unanimous vote in favour of the motion of President Noris Siliprandi to begin the annual Congress with an Antonini Lecture, forever. As reported below, the tradition began immediately at the Congress in Saint-Vicent, Italy, and is continuing. In this paper I report an account of the Eraldo Antonini Lectures that I attended over the years and until September 2019, a few months before the pandemics lock down.

## Incipit

**Fig. 1:**
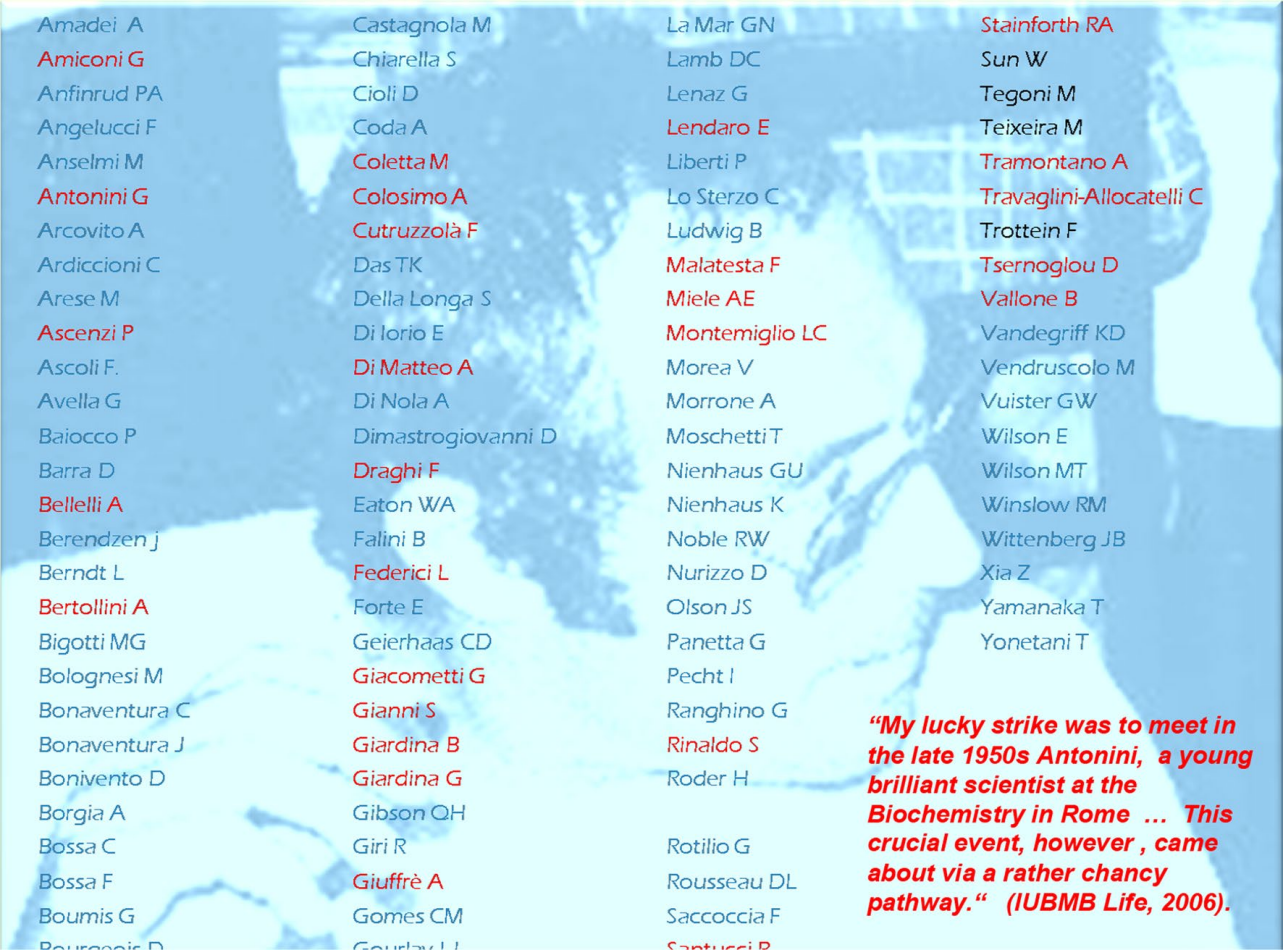
ANTONINI at a Conference in CAPRAROLA (IT)

NOMEN EST OMEN. Admittedly ERALDO is a rather uncommon name. According to the cabala, the bearer is supposed to be an intelligent cleaver flexible leader. As a scientist I am supposed not to believe horoscopes, yet in this case the prediction would seem to fit professor Antonini’s personality. He was a brilliant scientist and a charming leader that attracted outstanding visiting scholars, smart post docs and cleaver students from Italy and abroad. With a university degree in Medicine (1954), he achieved over a relatively short scientific career an undisputed international reputation. Antonini published over 300 papers in high quality scientific Journals and one book [1], delivered a large number of invited lectures at Conferences and was highly cited as witnessed by Nature (303, 114, 1983: *Italy’s top scientistst).*, The names of many of his collaborators are superimposed to his picture shown in Fig. 1.

The premature death of Antonini (18 March 1983) was a blow that stroke like an earthquake family, friends and many biochemists worldwide. An obituary by Brunori, Chiancone & Wyman was published in the Trends in Biochemical Sciences January 1984 [2]. Noris Siliprandi, professor of Biochemistry in Padova and President of the SIB (Società Italiana di Biochimica), proposed that the yearly Congress of the Society should always open with the Antonini Lecture. On September 26th 1983 at the first Congress after his death, I was asked to take responsibility for the lecture, and in spite of deep emotion, I accepted. In the Auditorium at Saint-Vincent, Italy, in front of a huge crowd of senior and younger Italian biochemists and after a warm welcome by my dear friend Jader Jacobelli, I began by recalling the extensive joint work on hemoglobin and myoglobin largely summarized in our book that was printed in 1971 [1]. I continued my presentation with an overall account of more recent work on the structure and function of fundamental hemeproteins involved in oxygen transport and metabolism, largely hemoglobin and cytochrome c oxidase; and illustrating new ideas and tantalizing enigmas originating from analysis of the experimental work. Eventually, I got to the end of what proved to be my most difficult talk, ever.

Since 1983 most of the SIB annual Congresses opened with the Antonini Lecture. For the list of these 33 Lectures visit the web site of the Department of Biochemical Sciences, Sapienza University of Rome: http://biochimica.bio.uniroma1.it/DSB/AntoniniLectures.htm

In addition, four memorial conferences were held in Rome starting 2003 on the XX anniversary of Eraldo’s death. Prof. Kurt Wüthrich accepted to come and deliver a *Lectio magistralis* in the Aula Magna of the Sapienza University.

This account presents comments and remembrances on a subset of the 33 Antonini Lectures and the four memorial Conferences between 1983 and 2019. I decided to limit myself to the events that I attended and those presented by colleagues that had direct contact either with Eraldo himself and/or with members of the Rome Group.

### 1984 Albert LEHNINGER in ISCHIA

In 1984 at the Congress in the island of Ischia, the Antonini Lecture was delivered by Albert LEHNINGER talking about Electron transport and proton translocation by cytochrome oxidase in its natural habitat. At the time Albert was a stellar figure in world’s Biochemistry for his science on the mechanism of energy transduction by mitochondria, and because of his spectacular textbook on Biochemistry [3] that revolutionized the teaching of our discipline worldwide. His lecture was very advanced and crystal clear as you may have expected from such an experienced Professor: he dealt with the variable stoichiometry between electrons delivered to oxygen and protons translocated in the respiratory chain, a crucial point for mitochondrial energy transduction. For many years the variability of the apparent stoichiometry had been a topic of harsh scientific debate possibly solved nowadays.

In the early sixties, Albert had been a visiting professor in the Biochemistry Institute at the invitation of Paolo Fasella, another brilliant pupil of professor Alessandro Rossi Fanelli, the founder of the Rome Biochemistry school. Lehninger and his wife Janet spent with us a few months with the desire to visit the Eternal City of course but also to discuss science, and work hard to complete his book on The Mitochondrion (published in 1964) [4]; he was writing “professionally” in a large quiet room on the second floor of the building. Naturally he met Antonini and even proposed to carry out a joint stopped flow experiment to investigate the kinetics of oxidation of reduced cytochrome c by ferricyanide; the experiment was clear cut and oxidation was determined to be very fast (rate constant around 10 + 9 M − 1 s − 1).

While Albert was in Rome, I was quite fortunate to have some opportunities to chat with him. The man was straightforward, very kind and easy to approach; discussions were very informal and he looked happy to listen and ready to give advice. I also became acquainted with his wife because they were both avid sailors and owners of a 40’ Sparkman & Stephens yacht kept harbored in Chesapeake bay and sailed to Bermuda. I drove them a couple of times to the harbor of Anzio to look at sail boats; the days were sunny and we looked carefully at a small 12’ dinghy I was dreaming to buy. It was a pleasant period spent with lovely people.

### 1985 Robert HUBER in RIMINI

In 1985 at the opening the SIB Congress in Rimini, Robert HUBER (Martinsried, Germany) delivered the Antonini Conference entitled: The structural basis of photosynthetic light reactions in bacteria. The 3D structure of the Reaction Center (RC) from *Rhodopseudomonas viridis *was the first membrane bound system ever solved. This extraordinary achievement [5] was the basis for conferring in 1988 the Nobel Prize in Chemistry to R. Huber, J. Deisenhofer and H. Michel from the Max Planck Institute of Biochemistry. It is well known that photosynthesis using energy from sunlight to make carbohydrates out of water and carbon dioxide, is the fundamental process of life. The conversion of light into chemical energy occurs via the RC, the key complex of several proteins intrinsic to special membranes in the cell. Transportation of electrons via a number of proteins is crucial to charge separation across the membrane. The photosynthetic RC from the bacterium *Rhodopseudomonas viridis* was purified and after many years successfully crystallized. Solving the 3D structure by X-ray diffraction revealed the main features essential to energy conversion, starting from the architecture of and connections between the four different subunits important to function. The RC contains several pigments which absorb light, the intrinsic cofactors being four bacteriochlorophyll b, two bacteriophaeophytin, two quinones and a ferrous ion. After excitation by light, a charge separation that spans the cell membrane is formed in a few hundred picoseconds, with a quantum yield of essentially one. The beauty of the high resolution structure allows to unveil the complexity of the electron transfer paths; extensive quantitative calculations were made possible thanks to the availability of the structure at atomic resolution.

As one of the early pioneers in protein crystallography, Huber knew of Antonini’s work on the function of hemoglobin and myoglobin. Supported by Professor Walter Hoppe, Robert engaged into the difficult project of solving the 3D structure of erythrocruorin (a monomeric myoglobin-like hemeprotein) purified from the larvae of the mosquito *Chironomus thummi thummi*. The project which began in the late fifties, took 10 years of work to solve the 3D structure of erythrocruorin which was published in 1970 in the Journal of Molecular Biology, [6]. In Rome we were interested in studying the ligand binding kinetics of erythrocruorin. Therefore, around the end of 1969, on the occasion of a visit to my good friend Jürgen Engel working at the at the MPI fur Eiweiss und Lederforschung in Goethe Str., Münich, I visited Huber’s Laboratory together with Jeffries Wyman (visiting professor in Rome). We had the privilege to be shown the hardware model of the *Chironomus* erythrocruorin and to be made aware of the similarities and differences with respect to horse myoglobin. As Huber stated, the fact that the overall fold of the oxygen carrying hemeprotein from an insect was very similar to that of mammalian myoglobin suggested, for the first time, a universal globin fold, a fundamental evolutionary principle nowadays generally accepted. After the Nobel, Robert continued to publish very important results driven by a great passion and success to develop structural biology,: “My fascination for protein structures and crystallography has never waned. Proteins are nice, crystals are nicer”.

### 1986 Bo G. MALMSTRÖM in MESSINA

In September 1986 at the SIB Congress in Messina the invited speaker for the Antonini Lecture was Bo MALMSTRÖM (Gӧteborg, SE), an outstanding biochemist/biophysicist, a leading authority in the field of the structure and function relationships in copper proteins, and chairperson of the Nobel Committee for Chemistry. In Messina, he delivered a talk on *Cytochrome c oxidase: an electron-transport driven proton pump*.

Bo was a fun-loving man, a good companion and serious musician. He was a sincere personal friend of Eraldo and his family; this friendship extended throughout the seventies and led to several visits to Rome by Bo, Betty and their children. Back in 1968, Bo visited the Institute in Rome for a couple of months to carry out with Eraldo and Alessandro Finazzi Agrò a study on the kinetics of electron transfer of laccase, a copper oxidase that had been studied extensively in Gӧteborg. The mechanism of the oxygen reduction by laccase proved to be quite intriguing [7] and paved the way to a simple yet profound concept indicating that only multi-electron entry into the enzyme led to a state capable of fast reaction with oxygen.

At the Congress Malmström illustrated a seminal and successful experiment on cytochrome oxidase carried out with the Rome group by Malmström himself and Colin Greewood, a visiting professor from Norwich. This sophisticated stopped flow experiment was published in 1970 as a Nature article [8]. Given that cytochrome oxidase catalyses the complete reduction of di-oxygen to water, we had to face a complex mechanistic problem to understand how a one-electron donor (reduced cytochrome c) is effectively coupled to a four-electron transfer to di-oxygen. The hypothesis that oxygen reduction by cytochrome oxidase may proceed via multi-electron transfer was consistent with the fact that cytochrome oxidase contains four electron accepting sites (Cyta, Cyta3,CuA and CuB). The idea was to compare the rapid reduction of the fully reduced enzyme with the oxidation rate of partially reduced state(s). After mixing oxidized cytochrome oxidase with sub stoichiometric concentrations of reduced cytochrome c in the presence of excess oxygen, the electron distribution in the enzyme was followed by transient spectroscopy. It was clear that the limited number of electrons from cytochrome c tended to reduce cytochrome a belonging to different cytochrome oxidase molecules; and electron redistribution among different partially reduced cytochrome oxidase molecules would become rate limiting. Thus it seemed unlikely that complete oxygen reduction may occur in four sequential one-electron steps. Malmström closed his Conference by recalling how this cooperative model for cytochrome oxidase was extensively discussed at the Manziana Conferences on metalloproteins, informal discussion meetings organized by Bruno Mondovì and collaborators.

### 1992 Carlo CROCE in PERUGIA

In September 1992 the opening Antonini Lecture at the 37th SIB meeting was presented by Carlo CROCE, professor of Medicine at the Thomas Jefferson University of Philadelphia and subsequently Director of the Comprehensive Cancer Center at the Ohio State University. A highly considered molecular oncologist, Professor Croce became widely known because of his discoveries emerging from studies on leukemias, lymphomas and other types of cancer. With an H index of 237 Carlo is one of the most cited scientists in the world.

The straightforward stimulating lecture he delivered in Perugia was entitled *Molecular genetics of human cancer*, *sic et simpliciter*. Carlo was at the time and still is one of the most successful Italian scientists worldwide because of several seminal discoveries such as: the juxtaposition between the genes of human immunoglobulin and the oncogene MYC and its deregulation in the Burkitt Lymphoma; the discovery and characterization of the gene BCL2 involved in the follicular lymphoma and the role of the genes ALL1/MLL and TCL1 associated with different types of leukaemia. His papers had an important role in the development of innovative methodologies for the diagnosis, prevention and treatment of cancer [9]. At the end of last century, Croce shifted his approach being interested in the role of microRNAs in oncology [10], and cloned many of these small oligomers involved in the control of cancer progression.

I feel that Prof. Antonini would have been particularly pleased to listen at the excellent conference of Carlo whom he knew since he was a medical student at the University of Rome. At the time, Carlo had been working for a couple of years at the Regina Elena Institute and the Biochemistry Institute where Eraldo had his laboratory and his group. Therefore he became acquainted with Eraldo who was attracted by the vivid intelligence and the talents of Carlo. I was told that in the late sixties, on the verge of deciding whether to emigrate to the USA, Carlo asked some advice to Eraldo who already had a wide reputation in Italy and abroad for his original scientific discoveries. He was encouraged to emigrate because, at the time, the scientific infrastructure necessary for the approach to cancer genetics that Carlo wanted to pursue was not available in Rome. A good decision in retrospect.

### 1995 Joe McCORD in TORINO

In 1995 in Torino the Antonini Lecture was delivered by Prof. J. McCORD from Denver (USA) formerly a brilliant student of Irwin Fridovich and first author of one of the most revolutionary discoveries in Biochemistry. In 1969 McCord and Fridovich discovered Super Oxide Dismutase [11] the copper-zinc enzyme catalyzing the dismutation of the superoxide radical with formation of hydrogen peroxide and oxygen, and unveiled its vast physiological significance:

$${\text{O2}} - + {\text{ O2}} - + {\text{2H}} + \to {\text{H2O2}} + {\text{O2}}$$

In a fascinating paper published in 2001 for the centenary of the JBC [12] Prof. Irwin Fridovich reviewed the saga linked to the unexpected discovery of this fundamental enzyme as it emerged from studies on the biochemistry of xantine oxidase and sulphite oxidation. I quote: *“To this day I recall the stunning impact of McCord’s data. Previous misconceptions were swept away, and it was immediately clear that xanthine oxidase was releasing superoxide into free solution. … inhibitors of cytochrome c reduction must be acting catalytically, and the only feasible way they could do so was by dismutating superoxide into hydrogen peroxide and oxygen. Bravo Joe McCord! εὕρηκα. … the Super Oxide Dismutase activity in bovine erythrocytes proved to be abundant and stable and it was soon purified. … Joe and I felt like kids in a toy shop.”* (McCord, J. M., and Fridovich, I. 1969, Superoxide dismutase. An enzymic function for erythrocuprein (hemocuprein). J. Biol. Chem. 244, 6049–6055 22). [11].

Indeed the title of McCord’s Lecture in Torino was Superoxide dismutase and the oxidant/antioxidant balance. Many people do not realize that the whole field of radical biology and pathophysiology which exploded over the last decades of the XX century came to birth in 1969 with the discovery of SOD catalyzing the quenching of superoxide, and thereby the chain of oxygen radicals involved in triggering cellular damage. The interest of the Rome Biochemistry group in the copper containing enzymes, championed by G. Rotilio, helped focus and extend the general significance of the discovery of SOD.

Understanding the mechanisms and physiological roles of superoxide radicals and of SOD unveiled the general significance of radical biochemistry in several physiological and pathological events, such as inflammation, phagocytosis and immune response. A basic unexpected event was the discovery that leukocytes and neutrophils produce a burst of superoxide radicals during phagocytosis, as shown by Bernard Babior. In a different context, the injury experienced by the heart during reperfusion following an ischemic event indicated that damage of the myocardium was to be attributed to a burst of oxygen radicals, changing the outlook of this important medical treatment. Thanks to Fridovich and McCord it is nowadays accepted that oxygen radicals biochemistry is involved in several medical aspects of aerobic metabolism. In my opinion, the discovery of SOD should have been seriously considered for the Nobel Prize in Medicine or Physiology given that it marked the birth of the whole new field of radical bio-medicine.

### 1999 John E. WALKER in ALGHERO

John Walker was awarded the Nobel prize in Chemistry in December 1997 with the following motivation: …”for elucidation of the enzymatic mechanism underlying the synthesis of adenosine triphosphate (ATP)." He started a scientific career with a PhD in Pathology at the University of Oxford in 1969. Afterword he moved abroad for good many years initially working in Minnesota (USA) and later at the Institut Pasteur in Paris. In 1974 he joined the Chemistry Division at the famous Laboratory of Molecular Biology in Cambridge (UK), and is still there. In 1978 John started working full time on ATP synthase, a crucial enzyme of the mitochondrial oxidative posphorylation machinery [13] John and his co-workers were engaged for a good many years in the quasi impossible frustrating job of obtaining suitable crystals of the ATP synthase purified from bovine heart mitochondria and from eubacteria. Crystallization of membrane proteins was known to be an “impossible task”, and many smart crystallographers had failed for years. I was told that over and over again John was encouraged by Max Perutz to continue with the work given that success would be highly rewarding. In fact the final goal of solving the 3D structure of the enzyme may reveal the detailed mechanism involved in the synthesis of ATP and the chemistry underlying the utilization of the trans membrane proton gradient. At the time Peter Mitchell had already published his chemiosmotic theory that changed the whole field and implied that the different components of the redox respiratory chain are pumping protons across the inner mitochondrial membrane creating a trans-membrane gradient driving ATP synthesis through ATP synthase.

When Prof Walker accepted the invitation to open the 44th SIB Congress in Alghero in September 2019, he was already a Nobel laureate. His conference was entitled: *ATP synthesis by rotary catalysis.* The 3D structure of the catalytic domain at 2.8 Å resolution had been solved in 1994 and the lecture was largely based on this outstanding success. He illustrated the difficult path that proved necessary to arrive at the 3D structure. Initially and for several years the work was focussed in establishing the subunit composition and the amino acid sequences of ATP synthase from different sources. Attention was concentrated on the large globular domain containing the catalytic sites which bind the nucleotides and face the interior of the mitochondrion. This globular multi-subunit domain started yielding true crystals that however proved very fragile and unsuitable for high quality diffraction. From 1983 to 1990 total attention was devoted to improve the quality of the crystals of the catalytic domain. A painstaking job that was eventually successful: in 1994 the structure at 2,8 Å was available and published in Cell [14].

In spite of the limited resolution, the overall architecture of the enzyme and the interactions between the various subunits (α3, β3, γ1) were clarified and proved to be the key to gain insight into the mechanism. The open-to-closed conformational changes of the β subunits were coupled to a rotary mechanism driven by the single central γ subunit: the rotation of the γ subunit changes the proportion of the various conformers of the three β subunits modulating the binding affinity for the nucleotides. Two additional cleaver experiments were revealing for the rotary mechanism promoting the ATP synthase as the prototype of a molecular motor. A stellar experiment was carried out by Yoshida Masasuki who was able to visualize by fluorescence microscopy of a labelled actin filament bound to the central γ subunit the rotation of the whole enzyme during catalysis. Wolfgang Jünge proposed that the rotatory motion was connected to the proton transport through the binding to carboxyl groups in the C subunits in the center of the whole structure*.*

### 2002 Giuseppe ROTILIO in PALERMO

At the SIB meeting in Palermo, September 2002, the opening lecture entitled *Redox regulation of apoptosis signalling,* was delivered by Joe ROTILIO, one of the outstanding members of the Biochemistry school founded by Rossi Fanelli. Joe received the University degree in Medicine from the University of Rome; while working for the experimental thesis he discovered a simple original method to purify amine oxidase, a copper protein from bovine serum. I remember that among the members of the Institute of Biochemistry this productive result by a student was considered very exciting.

Antonini had a high opinion of Rotilio as a scientist and later as a professor. In the early period of his career Joe devoted himself to the study of copper proteins with a considerable success. He was responsible for installing and operating at the Institute of Biochemistry the first Varian EPR facility in Italy, which proved essential to obtain fundamental information on the structure and configuration of metals bound to proteins.

Rotilio presented his approach to the role and function of the Cu–Zn superoxide dismutase. Joe had been working on erythrocuprein, a puzzling copper protein with unknown function when, in 1969 McCord and Fridovich discovered that erythrocuprein is an enzyme which catalyzes the dismutation of the superoxide oxide radical; thus they gave it the name of superoxide dismutase. Rotilio immediately understood that McCord and Fridovich’s fundamental discovery would pave the way to a novel important field centred on the role of free radicals in the homeostasis of the cell [15] In his Lecture Rotilio highlighted the explosion of interest for the role of superoxide and other radicals in physiology and medicine, and recalled that Prof. Antonini supported scientific programs and investments in the biochemistry and biophysics of the relevant copper proteins. He illustrated how an intracellular redox unbalance would act as a sensor, regulating nuclear transcription factors; and outlined the significance of the equilibrium between reduced and oxidized glutathione in modulating apoptosis signalling.

In the last part of his lecture Rotilio illustrated the correlations between the metabolism of metal ions and their effects on human nutrition and health. This was motivated by his prevailing interest in nutrition given his role as President of the National Institute for Nutrition. Joe outlined his ideas about human evolution and human nutrition [16] which he discussed in several interesting publications and books, along an original line of thoughts which elicited great interest.

### 2003 Kurt WÜTHRICH in ROME

On the XX anniversary of Eraldo’s death, a Memorial Conference was delivered by Kurt WÜTHRICH (ETH, Zürich), Nobelist in Chemistry 2002, who kindly accepted to come in spite of heavy commitments. His title was *NMR in Structural Biology and Proteomics*, presented in the Aula Magna of the University of Rome "La Sapienza" in the presence of the Deputy Rector and a huge crowd. Kurt was a friend and an estimator of Antonini since the late sixties. At the time he was working at the Bell Labs in Murray Hill at the dawn of the explosion of high resolution NMR to unveil the 3D structure of proteins in solution [17]. Like other smart biophysicists he was working with hemeproteins, notably with myoglobin whose 3D structure had been solved by John Kendrew and coll. Antonini was offered to collaborate with Kurt and Bob Shulman in an attempt to assign proton resonances in myoglobin by collecting NMR spectra of proteins reconstituted with different hemes: comparing the protoheme-containing wild type with myoglobins reconstituted with meso and deutero hemes. After preparing a substantial amount of the three reconstituted proteins, I flew to US and traveled to Murray Hill, successfully carrying the frozen material through the US Custom Office in New York by explaining why the famous Bell Labs needed this special material that could be prepared only in the holy city of Rome. The Custom Officer was satisfied with my explanation; and the 220 MHz NMR spectra collected by Kurt were more than satisfactory, clean and interpretable. A number of heme protons were unequivocally assigned being shifted away from the ocean of protons of the polypeptide chain; and two resonances were assigned to protons on the proximal histidine. Data showed that in determining the distribution density of unpaired electrons in the heme group, the protein moiety is more important than the heme substituents. The paper Shulman et al. was published in the PNAS, 1969 [18].

Over the following decades, Kurt was often in contact with people from the Rome Biochemistry group. We had frequent collaborations, in part related to the organization of biophysics EMBO schools in collaboration with Heini Eisenberg and Sandro Coda from Pavia; and to new initiatives concerning IUPAB business. In 2017 Prof Wüthrich kindly accepted the invitation to deliver a second Antonini Lecture, this time at the 59th SIB Congress in Caserta by the title: *NMR – from Basic Research to Daily Human Life*. It was on the last day of summer that he spoke in the lecture theatre in Caserta, which was absolutely packed with biochemists of all ages. The talk was crystal clear and very exiting; we were told about the latest NMR work on molecular neuropharmacology and the importance of discriminating among different brain receptors [19]. Professor Wüthrich is universally recognized as the scientist who pioneered the stellar progress of NMR to unveil protein’s 3D structure in solution [20], and their role in pathophysiology, creating the basis for significant progress in molecular Medicine; this was the motivation for the award of the Nobel in 2002.

### 2003 Harry B. GRAY in ROME

Believe it or not, in a short while it will be half a century since Harry Gray (Caltech) came to Rome for the first time, in the spring of 1973, to discuss science with Bruno Mondovì, a senior member of Rossi Fanelli’s Biochemistry group working on copper proteins. Bruno was a scholar and a gentleman, widely known for original contributions to the structure and function relationships in metalloproteins published with his smart young collaborators. Harry‘s visit led to the initiation of a series of informal discussion meetings on the Biochemistry and Biophysics of copper in living systems, that became known as the Manziana Conferences. A small number of brilliant scientists from outstanding institutions were invited to discuss informally, in the garden with blackboard and no time limit, the topic under debate. It was at Manziana that Harry came to know and appreciate Eraldo who was a regular participant not only because cytochrome-c-oxidase contains functionally essential copper but also because he had a special talent for kinetics.

No surprise that when the occasion came in December 2003, Harry was invited by Mondovì, Rotilio and Finazzi Agrò to deliver in Rome the Antonini memorial conference. His talk was a masterly overview on *The Currents of Life: Electron Flow through Metalloproteins* [21]. Harry is known to be an engaging speaker with a special talent to present a complex advanced scientific problem in a simple but rigorous vein. He began his conference by recapitulating Rudy Marcus theory of electron transfer, and moved from biological inorganic chemistry to mechanisms of electron transfer in proteins, highlighting that these reactions are key steps in photosynthesis, respiration, drug metabolism, and many other biochemical processes.

He presented paradigmatic examples of biological systems where electron transfer occurs between metals or metal-containing cofactors bound to a protein but separated by large distances (often greater than 10–15 A). Since the rate of electron transfer in proteins rapidly decreases with distance, Gray illustrated why electrons have to tunnel through a folded polypeptide to overcome long distances [22]. He recalled that by employing ruthenated iron and copper proteins, single-step electron tunneling can be modulated from nanosecond to microsecond, at distances up to 15/20 Å. When transport occurs over even longer distances, multistep electron tunneling architectures through intervening tyrosines and tryptophans, are necessary. The distance dependence of electronic coupling in a native protein is controlled by basic structural motif, helices, sheets and their tertiary arrangements. The conference at the University La Sapienza, was widely appreciated by the audience, including Eraldo’s family members.

### 2009 Emilia (Milina) CHIANCONE in CATANIA

Milina has been for twenty years a very close and extremely valid collaborator of prof. Antonini. In 1961 she moved from Milan to Rome to work in the Institute of Biological Chemistry directed by Prof A. Rossi Fanelli. As a member of Antonini’s group, she was assigned the task to run the Beckman Spinco Ultracentrifuge invented by T. Svedberg, the most advanced instrument of the time to assess the hydrodynamic properties of macromolecules. The sedimentation coefficient and the diffusion coefficient allow to obtain an estimate of the molecular weight of the protein of interest; in the specific case hemoglobin, the multimeric protein of interest to Antonini and Jeffries Wyman, just arrived from Paris as a visiting professor at the Regina Elena Institute for cancer research. In 1962 Milina went to Birmingham, UK, to work with Geoffrey and Lilo Gilbert, leaders in that area of Biophysics. Once back to Rome Milina was by definition the national expert in ultra-centrifugation [23] and began producing very interesting papers on the association-dissociation equilibria of tetrameric human hemoglobin and later hemoglobins from other species. Along this line of research she achieved with time a leadership that was widely recognized; suffice to recall the extremely intriguing case of the hemoglobins from *Scapharca inequivalvis* that she investigated in collaboration with Franca Ascoli. After determination of the crystallographic 3D structure, the homodimeric Scapharca hemoglobin became the paradigm for allosteric cooperativity in a homodimeric oxygen carrier [24].

When in 2009 she was asked to deliver the Antonini Lecture at the SIB Congress in Catania, she decided to concentrate on a totally new topic that she successfully explored over the last decade in collaboration with Simonetta Stefanini e Pierpaolo Ceci. The tile of her conference was: “*Dps proteins, an efficient means to detoxify iron and protect DNA in the bacterial response to oxidative stress”.* The project on DPS proteins was a follow up of the work that Milina and collaborators had carried out for several years on the metabolism of iron, the structure–function relationships in the ferritin superfamily, and the medical significance of dis-function(s) related to oxidative stress. DPS are bacterial proteins belonging to the ferritin superfamily, that are induced in microorganisms as a response to oxidative or nutritional stress; thereby they are components of a complex defence system gyred to protect DNA [25]. DPS have some similarities with ferritins but also distinct structural properties; the first DPS identified in *Escherichia coli* is a structural analogue of the ferritn from *Listeria innocua* that protects DNA against oxidative damage either by physical association or by quenching the toxic combination of Fe(II) and hydrogen peroxide. The interest of these iron containing proteins increased considerably once it was shown that they are required for virulence in *Salmonella enterica*. The audience in Catania expressed appreciation for the lecture and recognized the quality of the science and the exceptional clarity of the presentation.

### 2012 Maurizio BRUNORI in CHIETI

I was invited by Luca Federici to deliver the opening Antonini lecture at the 56th SIB Congress held in Chieti. The title of my talk was: *Form and substance: morphogenesis of a protein,* a theme that has acquired considerable medical relevance once it was established that protein misfolding is a crucial step in the onset of several devastating neurodegenerative disorders.

In the mid sixties the protein folding problem had been of great interest for the Rome group because of two unrelated events. The first was a cleaver purification protocol invented by Professor Antonini to obtain pure myoglobin from the radular muscle of *Aplysia*, a Mediterranean mollusc. In 1957, working at the Zoological Station in Naples, he decided out of the blue, to boil the homogenate of the muscle after centrifugation; all proteins precipitated except the myoglobin, which could be collected in the supernatant and proved to undergo a fully reversible thermal denaturation, contrary to horse or whale Mb which upon heating coagulate. The second event was the decision of Charles Tanford to spend a period in Rome while writing a review on folding for Advances in Protein Chemistry. Starting from Anfinsen’s experiment, daily discussions with Tanford, Lumry and Wyman triggered interest and fostered the start a project on the denaturation of *Aplysia* myoglobin. Our first paper was published in 1968 in the J Mol Biol [26], and paved the way to the experimental thesis of Giorgio Giacometti. Unfortunately we failed to realize that denatured Aplysia Mb at 80 °C, keeping a substantial fraction of alpha-helix was a good example of a molten globule intermediate, a state later “discovered” and defined by A. Wada and O. Ptysin.

After a period in the eighties when working on protein folding was fairly unpopular, the whole field resuscitated with novel ideas starting from the funnel theory. I presented to the audience in Chieti the highlights of the field and the original concepts that emerged from the understanding of the role of protein misfolding in medicine. Assessing the structure of the folding/unfolding transition state correlating mutagenesis and kinetics according to the so-called F-value analysis introduced by Alan Fersht has been a revolution; Eraldo would have been excited to engage in the determination of the structure of a transition state using stopped flow kinetics, his preferred toy. An important step forward originated from understanding the correct significance of the amyloid fibrils and their role in pathophysiology of neurodegenerative diseases [27]. I was convinced by Chris Dobson’s statement that in principle any protein can form an amyloid, the hallmark of a neurodegenerative disease such as Alzheimer, Parkinson, ALS and many others.

### 2013 Christopher DOBSON in ROME

Christopher DOBSON (Cambridge, UK) delivered a Lecture entitled *New Approaches to Understanding and Preventing Neurodegenerative Diseases* on 19 March 2013, the XXX anniversary of Antonini’s death. Chris had been working on the molecular mechanism connecting protein misfolding and formation of amyloid fibrils in the brain, considered the hallmark of a number of fatal neurodegenerative diseases such as Alzheimer’s and Parkinson’s. Given the great importance of this topic, a huge crowd filled the Aula Magna of the Sapienza University.

A large group of human diseases arises from the failure of a specific peptide or protein to achieve its characteristic functionally competent native conformation. The accumulation of misfolded states is associated to aggregation into highly ordered fibrillar structures called amyloid, a term coined in 1853 by Rudolf Virchow. Work in vitro established that many different proteins may form, under proper conditions, fibrillar aggregates that have some commonality in overall architecture. Chris proposed that formation of amyloid is an inherent property of most proteins, involving as it does interactions among β-sheets normally buried inside the native fold; naturally the propensity to achieve an amyloid state can vary dramatically with sequence [28]. Aggregation leading to fibrils can be initiated starting from different states such as denatured, intermediate or native; the message is that a clear understanding of the misfolding cascade would pave the way to the possibility to treat some of these devastating diseases.

A crucial task has been considered the understanding of the microscopic kinetic steps involved in aggregation, a very complex problem given the large number of interconverting species via a multitude of processes, including heterogeneous nucleation and secondary nucleation. By dissecting the microscopic kinetic steps involved in aggregation of Aβ42, the peptide responsible for the extracellular amyloid accumulating in Alzheimer’s disease, one may hope to select or design specific inhibitors that may interfere with aggregation [29].

Chris clarified that in spite of clear clinical differences, different neurodegenerative diseases have some fundamental commonalities. The world population is associated to an increase in the number of older people and these diseases are strongly age-related. Alzheimer's disease alone may affect up to one-half of people over 85 years and the number of Alzheimer's patients is anticipated to increase to around 140 million by 2050. Given that no therapy is presently available for these fatal disorders, a forthcoming neurological disaster will have a devastating impact on individuals, families, caregivers and societies at large. The economic burden may rise to about one trillion US$ per year in the USA alone. The magnitude of the problem calls for a broad responsible initiative and a strong global commitment to fundamental research in order to discover an effective necessary therapy.

Because of his fundamental scientific discoveries, Professor Dobson was awarded in 2014 the International Feltrinelli Prize for Medicine by the Accademia Nazionale dei Lincei. He presented an overview of the scientific and socio-economic problem linked to neurodegenerative diseases in the presence of President Giorgio Napolitano during the opening session of the Lincei.

### 2013 Jean-Pierre CHANGEUX in FERRARA

For the 57th SIB Congress in Ferrara (September 2013) the Committee invited J-P CHANGEUX to deliver the opening Antonini Lecture entitled: *Allosteric properties of pentameric receptor-channels: from bacteria to brain.* Soon after his PhD, Changeux was already known worldwide because while working on l-threonine deaminase in the late fifties he had the intuition that enzyme activity can be regulated by chemical interactions at sites distinct from agonist binding sites. This led to the general concept of allostery which was formalized in the famous paper that he published in collaboration with Jacques Monod and Jeffries Wyman in may 1965 [30]. When Eraldo Antonini flew to Paris sometimes around the end of that year invited by Jacques Monod to deliver a talk on hemoglobin –the paradigmatic allosteric protein-, he obviously met Changeux; but unfortunately I had no report of their scientific conversations.

The outstanding talent of Jean-Pierre Changeux emerging from analysis of half a century of intense scientific engagement, has the mark of an original mind. Very early after PhD, Changeux had the intuition that the function of membrane proteins involved in synaptic transmission were modulated through allosteric conformational changes. The obvious consequence of such a hypothesis, if proven to be correct, would be that neurotransmitters and drugs active in the brain would operate via the same regulatory mechanism. Thus he engaged on a very ambitious fundamental problem in neuroscience focused on brain receptors and started with the isolation and purification of the nicotinic receptor for aceylcholine (nAChR) and continued with its first visualization by electron microscopy, the demonstration of its pentameric structure, the determination of its amino-acid sequence and complete cloning, and eventually the determination of its 3D structure by X-ray crystallography and molecular dynamics simulations [31]. A project that lasted several decades and yielded a breakthrough with the multi-scale understanding of the human brain: from the microscale of receptors and synapses (allostery, receptors, epigenesis) to the macroscale of networks and consciousness (global neuronal workspace).

The introduction of the concept of allosteric modulation has created a paradigmatic change in pharmacology with the discovery of a novel category of agents shown to bind to sites distinct from the classical orthosteric binding site for neurotransmitters and/or hormones and referred to as allosteric modulatory sites. Changeux’s work revolutionized our understanding of the brain and its pharmacology, opening myriads of clinical discoveries in neurology and psychiatry, from drug addiction, cognitive enhancement, ageing and schizophrenia.

### 2015 Ada YONATH in URBINO

In 2015 the SIB Congress was organized by the University of Urbino and the opening lecture delivered by Ada YONATH (Weizman Institute, Israel) was entitled*: Resistance to Antibiotics and Preserving the Microbiome*. It was an hour of joy and excitement to listen at Ada unfolding for the audience the incredible story of the discovery of the 3D structure of the ribosome, an intricate complex of proteins and RNA, huge in size, very flexible, devoid of internal symmetry, features that all conspire in making preparation of suitable crystals very difficult indeed [32].

Ada decided to tackle this incredibly complex problem using the tools and methodologies proper to Biochemistry, purifying ribosomes from different organisms including extremophiles. For a good many years she worked hard in spite of the discouraging opinion of many scientists. She wrote: *“This was the beginning of a long quest that took over two decades, in which I was met with reactions of disbelief and even ridicule in the international scientific community. I can compare this journey to climbing Mt. Everest only to discover that a higher Everest stood in front of us”*. Fortunately some intelligent visionary scientists such as HG Wittmann and M. Sela continued to encourage Ada and provide financial support.

In the early to mid-1980s she published important results [32]. The visualization in the larger ribosomal subunit of a tunnel representing the path through which the protein being synthesized was progressing; the crystallization of the organelle from *Geobacillus stearothermophilus*; and the application of cryo-crystallography to reduce damage of the delicate crystals by X ray flux. I remember the passionate account she gave me after a SAC meeting at the EMBL outstation in Hamburg during a car transportation to the airport.

At the end of the 1990s the resolution barrier was broken, and in 2009 she was awarded the Nobel in Chemistry shared with T. Steiz and V. Ramakrishnan. The mechanism of cooperation between RNA and the proteins emerged very clearly, and new medically important aspects dealing with the discovery of new molecules to combat antibiotic resistance were illustrated. I believe that Eraldo and Ada never met, but I am sure that if Eraldo were to be in Urbino that day, he would be enchanted.

### 2017 Michael WILSON in ROME

For the XXXV anniversary of Eraldo’s death March 2017, the memorial conference was held at the University of Rome La Sapienza by Michael WILSON (Colchester, UK) who spoke about *“Serendipity and imagination in science: Eraldo Antonini and the early work on cytochrome oxidase”*. Mike’s title and his talk could hardly have been better suited to highlight the ingenious approach of Eraldo in tackling a scientific problem.

After his PhD on cytochrome c obtained at the University of Norwich working in Colin Greenwood’s Lab, Mike came to Rome as a post doc fellow. He arrived in February 1971 with his wife Angela and the few months old twins Emma and Ruth; and in 1973 he moved back to UK where he was offered a position in the Department of Chemistry at the University of Essex.

Mike is an extremely talented scientist and a wonderful human being, full of humour and very friendly with people. He has come back to the Rome Lab many many times even after Eraldo passed away. His contribution to the understanding of the relationships between structure and function in hemeproteins has been superb all around. Mike has been an active member of the group, driving people with enthusiasm and intelligence. I remember well when he was physically present in the kinetics Lab that special afternoon when the oxygen pulsed state of cytochrome oxidase was discovered thanks to a sophisticated double mixing stopped flow experiment [34]. The discovery of a substantial increase in enzyme activity was immediately apparent from examination of the sequence of stopped flow shots obtained under different conditions; the data recorded on a film and analysed by painstaking calculations, were clear-cut and the controls consistent.

This discovery allowed to rationalize a significant number of previous observations on the resting cytochrome oxidase, and led to a paper that was communicated to PNAS by Jeffries Wyman; indeed this was the first among several other publications including one that applied the MWC allosteric model to cytochrome oxidase and its proton pumping function [35]. The discovery of pulsed oxidase was communicated to a USA Gordon conference by Helmut Beinert, a guru of the oxidase, who had received the preprint in advance. We were informed that he was very positive about the novel result, and those that have known the aplomb of Helmuth may realize why we were so happy.

### 2019 Sir John WALKER, a second Antonini Lecture in LECCE, 14/18 September

The fantastic progress in understanding the mechanism underlying the ATP synthesis (see above, Lecture in 1999) paved the way to more extensive studies on the functional properties of the ATP synthase [36]. These additional discoveries at a higher degree of complexity represented the topic of the Antonini lecture assigned (once again) to Sir John WALKER as the opening event of the 60th SIB Congress held in Lecce.. The Lecture was chaired by Ferdinando Palmieri who had established years before a very productive collaboration with Walker’s group in Cambridge to prepare suitable crystals of some of the metabolites carriers of the mitochondrion. The title of the talk was*: “Regulation, functional analysis and assembly of dimeric ATP synthases in mitochondria”.*

The focus of the lecture was on the role of the supra-molecular structure in controlling proton uptake from the mitochondrial matrix at the level of the inlet channel, and in shaping the cristae. The structure of the dimer of the bovine ATP synthase solved by cryoEM, revealed the three fundamental rotatory states basic to the mechanism of catalysis involved in the ATP synthesis. The membrane domains embedded in the inner mitochondrial membrane of the organelle are in contact via interactions involving all domains of each monomer, the 3 helices of the β-subunits interacting with the so called supernumerary subunits (e, f and g); internal voids are filled by three cardiolipin molecules and other phospholipids.

A fundamental aspect of ATP synthase catalytic cycle is the control of the proton pathway which demands a set of stereochemically organized positively and negatively charged amino acids [37]. The clearly defined inlet half-channel passing through the a-subunit is negatively charged; while the outlet half-channel is more open funnel-shaped. The two are separated by a positively charged region. This highlights a structural theme common to several proteins involved in proton translocation. The so-called water wire is built by a string of water molecules in a hydrophilic cavity lined with polar amino acids; whose thermal fluctuations modulate proton transfer by rocking the water molecules. Following the so-called Grotthuss mechanism the string of water molecules makes transfer of protons most efficient even in neutral solutions.

During catalysis the monomer–monomer interfaces change their conformation to accommodate rocking motions. The overall architecture of the dimer appears to define an angle yielding a Y-like shape. The structures also suggest how the dimeric ATP synthases might be interacting with each other to form the characteristic rows along the tips of the cristae, moulding themselves to the range of oligomeric arrangements observed by tomography of mitochondrial membranes. Perturbation of the interface between dimers by site directed mutation affects the morphology of the cristae.

## Epilogue

Walker’s lecture in Lecce was the last conference held in front of an audience of several hundred people. That day of September we enjoyed the fantastic conference delivered by Sir John, sitting down as part of a huge crowd of friends, junior to senior biochemists, from all over Italy and abroad. No one could have imagined the global disaster that was just about to strike: in January/February 2020 the COVID 19 pandemics exploded worldwide producing about 6 million deaths still incresing, and a strict lock down was imposed more or less everywhere. Obviously no Antonini Lecture in presence of an audience has been held since that day.

## Data Availability

Not applicable.
